# The spleen tyrosine kinase (SYK): A crucial therapeutic target for diverse liver diseases

**DOI:** 10.1016/j.heliyon.2022.e12130

**Published:** 2022-12-07

**Authors:** Yaping Zhao, Rongrong Liu, Miaomiao Li, Pengfei Liu

**Affiliations:** a National & Local Joint Engineering Research Center of Biodiagnosis and Biotherapy, The Second Affiliated Hospital of Xi’an Jiaotong University, Xi’an, China; b International Joint Research Center on Cell Stress and Disease Diagnosis and Therapy, The Second Affiliated Hospital of Xi’an Jiaotong University, Xi’an, China; c Shaanxi Provincial Clinical Research Center for Hepatic & Splenic Diseases, The Second Affiliated Hospital of Xi’an Jiaotong University, Xi’an, China; d Department of Regenerative Medicine, School of Pharmaceutical Science, Jilin University, Changchun, China; e Key Laboratory of Environment and Genes Related to Diseases, Xi’an Jiaotong University, Ministry of Education of China, Xi’an, China

**Keywords:** Spleen tyrosine kinase (SYK), Liver diseases, Small molecule inhibitor, Therapy target

## Abstract

Spleen tyrosine kinase (SYK) is an enigmatic protein tyrosine kinase, and involved in signal transduction related with lots of cellular processes. It’s highly expressed in the cells of hematopoietic origin and acts as an important therapeutic target in the treatment of autoimmune diseases and allergic disorders. In recent years, more and more evidences indicate that SYK is expressed in non-hematopoietic cells and effectively regulates various non-immune biological responses as well. In this review, we mainly summary the role of SYK in different liver diseases. Robust SYK expression has been discovered in hepatocytes, hepatic stellate cells, as well as Kupffer cells, which participates in the regulation of numerous signal transduction in various liver diseases (e.g. hepatitis, liver fibrosis and hepatocellular carcinoma). In addition, the blockage of SYK activity using small molecule modulators is considered as a significant therapeutic strategy against liver diseases, and both hepatic SYK and non-hepatic SYK could become highly promising therapeutic targets. Totally, even though some critical points about the significance of SYK in liver diseases treatment still need further elaboration, more reliable biotechnical or pharmacological therapy modes will be established based on the better understanding of the relationship between SYK and liver diseases.

## 1 Introduction

Spleen tyrosine kinase (SYK) is a cytoplasmic non-receptor tyrosine kinase and has a central role in the regulation of various signal transduction. Even though the gene of SYK is first designated using a spleen cDNA library, this kinase is mainly expressed in the cells of hematopoietic origin, and effectively transmits different signals from T-cell antigen receptors (TCR) and B-cell antigen receptors (BCR). It binds to the phosphorylated Immunoreceptor Tyrosine Based Activation Motif (ITAM) and mediates inflammatory cascade. In recent years, SYK has also been demonstrated to express in non-hematopoietic tissues (e.g. vascular smooth muscle, normal mammary gland and pulmonary tissue) [1, 2, 3, 4]. Therefore, diverse biological functions, such as immune recognition and inflammatory response, can be effectively regulated by SYK, and SYK is also considered as a novel therapy target in lots of non-hematopoietic diseases (e.g. liver fibrosis, lung cancer, and diabetic cardiomyopathy) [5, 6, 7]. Currently, SYK inhibitors have been used in the treatment of rheumatoid arthritis, systemic lupus erythematosus, and other diseases, and several compounds show promising therapeutic action in clinical studies [3, 8]. Especially, SYK inhibitors are also regarded as functional tumor suppressors, and hold great potential in chemotherapy. Fostamatinib (R788) is the first SYK inhibitor tested in clinical phase I/II study for the treatment of B-cell lymphomas, and shows effective therapeutic action, indicating that SYK inhibition could be a novel therapeutic approach in cancer treatment [9, 10]. Besides, other SYK inhibitors, such as Entospletinib (GS-9973) and Cerdulatinib (PRT062070), are also identified as effective anti-cancer drugs in both pre-clinical and clinical trials [9, 11, 12].

In recent years, scientists notice that SYK is expressed in hepatocytes and hepatic stellate cells, even expressed in Kupffer cells [7, 13]. Therefore, the relationship between SYK and liver diseases attracts scientists' attention besides the multifactorial role of SYK in immune diseases. Moreover, based on the function of SYK in different biological processes and signaling pathways, most liver diseases, such as liver fibrosis and liver cancer, are relevant to SYK activity directly. The suppression of SYK activity abrogates hepatic neutrophil infiltration and resident immune cell activation, suggesting the important potential of SYK in immune cell-driven liver inflammation, liver steatosis, as well as liver cell death [14]. Thus, the current studies highlight SYK as a promising therapeutic target in the treatment of liver diseases [15, 16]. In this review, we mainly provide a general overview of the function and significance of SYK in the development of different liver diseases, and discuss the opportunities of SYK inhibition as novel therapeutic approach for liver disease treatment.

## 2 Characterization and origin of SYK in liver

**Figure 1:**
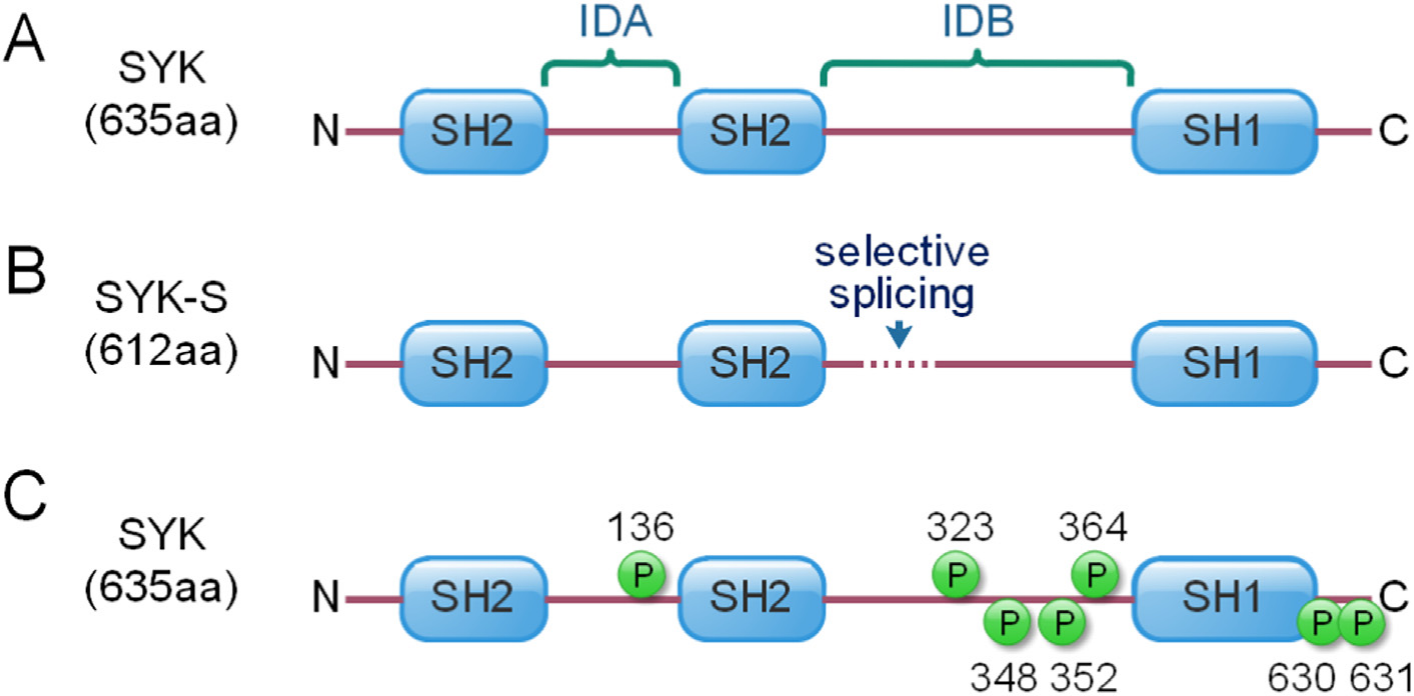
Structure of spleen tyrosine kinase. A. the structure of full-length SYK. B. the structure of short isoform of SYK protein (SYK-S). Because of the selective splicing during transcription process, compared with full-length SYK, SYK-S holds 23 amino acids less in the IDB domain. C. The key Tyr phosphorylation sites in SYK protein.

SYK is a member of the Zeta-chain associated protein kinase 70 (ZAP-70), and the gene of SYK is located at 9q22.2 in the human chromosome. Human SYK protein contains 635 amino acids with the molecular weight of 72KD. SYK consists of one tyrosine kinase domain (SH1) at C-terminal and two SYK homology 2 domains (SH2) at N-terminal. These three domains are linked together via two linker regions: interdomain A (IDA, the linker between two SH2 domains) and interdomain B (IDB, the linker between two SH2 domain and SH1 domain) [1, 17]. In addition, there is another short isoform of SYK protein (SYK-S), which lacks 23 amino acids in the IDB because of the selective splicing during the process of DNA transcription (Figure 1A and 1B). The activity of SYK is determined by the phosphorylation of tyrosine, and SYK-S also has less phosphorylation sites than SYK. However, compared with SYK, the functional defect of SYK-S in immune regulation is mainly caused by the weaker binding to ITAM, not the loss of crucial tyrosine phosphorylation sites (e.g. Tyr-323, Tyr-348 and Tyr-352) (Figure 1C) [18, 19, 20, 21]. Therefore, the significance of different phosphorylation sites still needs further investigation. In addition, mouse SYK protein contains 629 amino acids. The amino acid sequence and protein domains of mouse SYK are similar to those of human SYK, and the percent identity is 92.13%. Currently, five mutated form of SYK (S550Y, S550F, P342T, A353T and M450I) has been identified in human beings, and the variants cause increased phosphorylation and enhanced downstream signaling of SYK, resulting in immune dysregulation as well as systemic inflammation [22].

In recent years, many intracellular pathways are relevant with SYK activation. Usually, SYK binds to ITAMs in receptor-associated adaptors or in the tail of receptor chain, and mediates the ITAM-based signaling pathways. For example, SYK is involved in IgE/FcεRI signaling pathway via interacting with adaptors LAT1 and LAT2, and regulates the development of allergic diseases. In addition, the direct interaction between FcγRIIA and SYK without other adaptors also renders a strong downstream intracellular signal in platelet activation [1, 2, 23]. Therefore, the intracellular pathways related with SYK can be divided into adaptor-dependent signaling pathways (e.g. BCR, TCR, FcεRI and CLEC4E signaling pathways) and adaptor-independent signaling pathways (e.g. FcγRIIA, CLEC2, CLEC7A and CLEC9A signaling pathways). Currently, the main ITAM-containing adaptor proteins related with SYK are FcRγ and DAP12 in myeloid cells [2]. However, whether those adaptor proteins are still essential in hepatocytes remains unclear. Recently, scientists discovered another SYK-related ITAM-containing adaptor, 3BP2. Both 3BP2 and SYK correlate with severity of steatohepatitis, indicating that functional adaptor protein for SYK could be varied in different tissues [24].

The SYK in liver can be divided into hepatic SYK and non-hepatic SYK. Even though SYK is mainly expressed in hematopoietic cells and immune cells, scientists have confirmed that most hepatic cells also express SYK effectively. For example, SYK can be expressed in hepatocytes, hepatic stellate cells (HSCs), Kupffer cells as well as liver sinusoidal endothelial cells. The expression of SYK is upregulated in hepatocytes and HSCs during the process of liver fibrosis [7, 21]. On the other side, blood can be stored in liver and the blood volume can also be effectively regulated by liver to maintain proper physiological function [25]. Thus, there are kinds of blood cells in liver (e.g. lymphocytes and macrophages), which are considered as the non-hepatic origins of SYK. Especially, some studies have indicated that the splenic lymphocytes can migrate into liver, and regulate the development of liver disease, in which SYK may also play an important position [26, 27].

Even though some groups have demonstrated that SYK can express in various normal tissues, such as liver, kidney, heart and lung, the activity of SYK in spleen is much higher than other tissues, which is consistent with the transcription level of SYK [28, 29]. In recent years, the important position of spleen in the development of liver diseases have been revealed by different groups [27, 30, 31]. Splenectomy is also considered as an effective therapy method for the patients with hepatocellular carcinoma, hypersplenism or liver cirrhosis [32, 33, 34]. For example, splenectomy provides significant protection against liver fibrosis via activating ERK1/2 signaling pathway and suppressing JNK/TGF-β1 signaling pathway in hepatic macrophages, and the ratios of some immune cells in different phenotype are also affected in liver by splenectomy, which is related with the therapeutic action against liver diseases [34, 35, 36]. In addition, solid tumors usually contain large numbers of monocyte-derived macrophages which further promote the tumor progression, and monocytes also accumulate in the spleen during the process of tumorigenesis. More evidences indicate that the spleen monocytes can enter the solid tumors and contribute to the tumor infiltrating monocyte population in some degree, clarifying the key position of spleen in the process of cancer development [37, 38, 39]. Some studies have indicated that the inhibition of SYK activity can mitigate the acquisition of inflammatory phenotype in myeloid cells [40, 41, 42]. Therefore, the non-hepatic SYK, such as the SYK in spleen monocytes, could be an important therapeutic target for liver diseases, and the therapeutic action of splenectomy against liver diseases may be also relevant with the inhibition of non-hepatic SYK.

## 3 The relationship between SYK and different liver diseases

**Figure 2:**
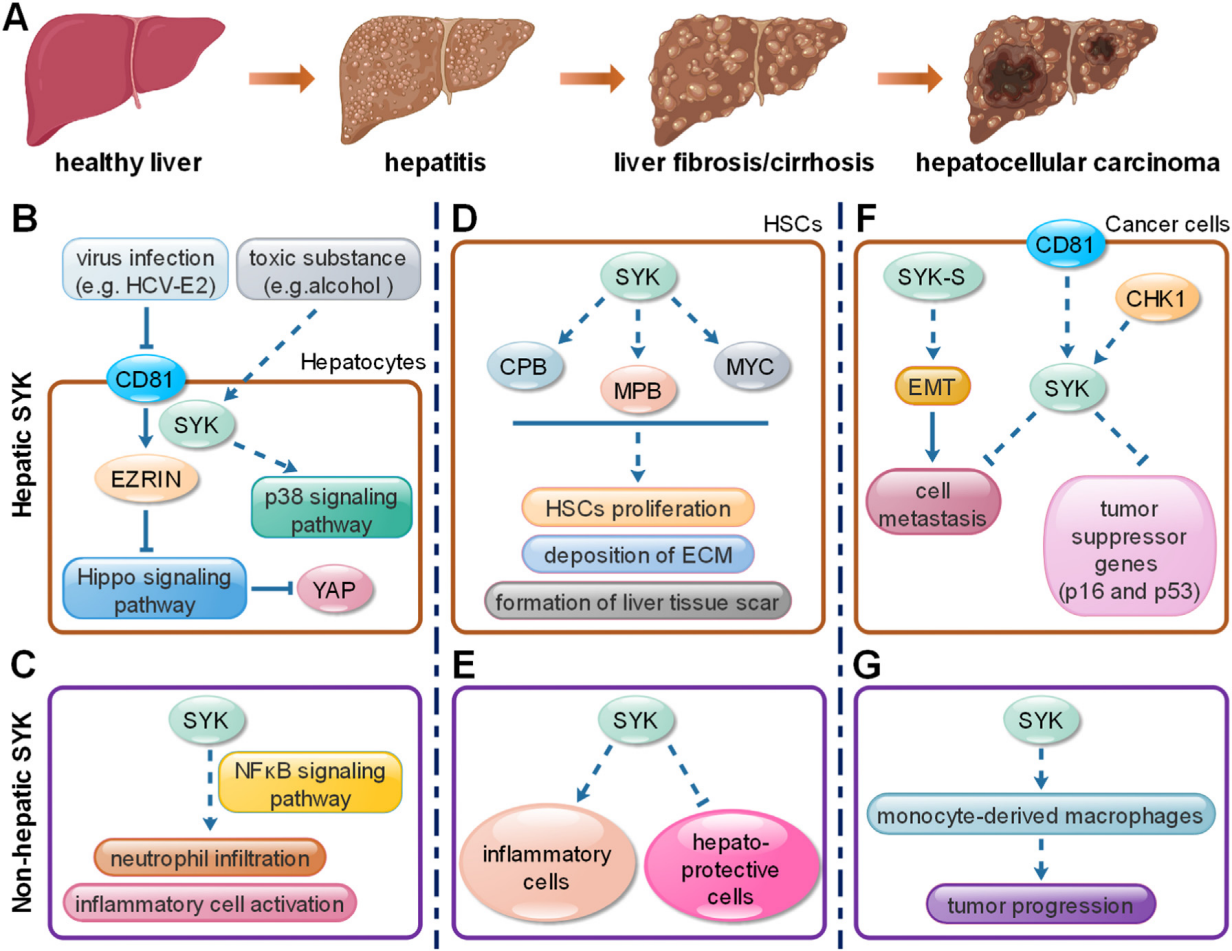
The relationship between SYK and different liver diseases. A. the process from healthy liver to hepatocellular carcinoma. SYK in liver can be divided into hepatic SYK (B, D, F) and non-hepatic SYK (C, E, G). The position of SYK in hepatitis (B-C), liver fibrosis/cirrhosis (D–E) and hepatocellular carcinoma (F–G) are present herein respectively.

SYK is related with various diseases, and participates in the regulation of numerous signal transduction in liver. For example, SYK is activated gradually during the progression of alcoholic or viral liver diseases, and therapeutically blocking SYK function by different chemical inhibitors significantly diminished alcohol-induced hepatic steatosis and viral hepatitis [7, 41, 43]. Moreover, the process of liver fibrosis is also accelerated by SYK activation, which promotes the development of liver cirrhosis and liver cancer eventually [7, 44]. Therefore, the activity of SYK in liver is positively correlated with the disease severity (Figure 2A).

### 3.1 SYK and hepatitis

Hepatitis, the inflammation of liver, can progress to liver fibrosis and cirrhosis, even liver cancer. Currently, hepatitis is mainly caused by hepatitis virus infection (e.g. Hepatitis A-E Virus) and toxic substances (e.g. alcohol and some hepatotoxic drugs). In recent years, the relationship between SYK and hepatitis have been investigated by different groups. Compared with normal human liver, the mRNA expression of SYK is significantly increased by 7.52-fold (HBV infection), 2.19-fold (HCV infection), 1.96-fold (alcohol abuse) and 1.34-fold (non-alcoholic steatohepatitis), indicating that SYK plays a critical role in the pathogenesis and development of both virus infection-induced hepatitis and toxic substance-induced hepatitis [7, 45].

In virus infection-induced hepatitis, scientists have confirmed that the expression of SYK and cytokines in hepatocytes can be significantly upregulated by Hepatitis B Virus (HBV) or Hepatitis C Virus (HCV) infection, and the increased cytokines and SYK expression in hepatocytes further enhance the transcription of fibrosis-related genes in hepatic stellate cells (HSCs), the core participants in liver fibrosis [7]. Therefore, SYK plays an important role in the process form viral hepatitis to liver fibrosis, and it’s considered as an important therapeutic target for different viral hepatitis [43, 46]. For example, the glypican-3 or E2 protein on HCV can bind to CD81 (the key receptor for HCV) and affect phosphorylation of Ezrin via SYK, leading to the activation of the Hippo signaling pathway and inhibition of yes-associated protein (YAP) signaling pathway in human hepatocytes [47, 48, 49]. The disruption of HCV engagement of CD81 also blocks the activation of SYK [46], which could become a novel anti-HCV strategy (Figure 2B). However, the NS5A protein of HCV also can strongly interact with SYK and suppress the kinase activity, indicating the multiple relationships between virus infection and SYK activation [50].

Alcoholic hepatitis in the most common toxic substance-induced hepatitis all over the world. The activation of SYK can be induced by alcohol, and SYK phosphorylation also joins in the regulation of immune cell-driven liver inflammation and holds a central modulatory position in multiple proinflammatory signaling pathways in alcoholic hepatitis [40, 41, 51, 52]. Therefore, the effective inhibition of SYK activation is also considered as a novel strategy against alcoholic hepatitis. Researchers have demonstrated that the inhibition of SYK can abrogate hepatic neutrophil infiltration and activation of resident immune cells induced by alcohol, and diminish alcohol-induced hepatic steatosis and apoptosis via NFκB signaling pathway, indicating the potential of SYK as a therapeutic target in human alcoholic steatohepatitis treatment (Figure 2C) [41, 53]. On the other side, the positive correlation also can be found between SYK expression and the pathogenesis of non-alcoholic steatohepatitis (NASH), and the administration of SYK inhibitor (R406) with PLGA nanoparticles is regarded as a potential therapeutic approach against mouse NASH [40]. Recent studies indicated SYK regulates the alternative p38 activation, which could be the important mechanism in toxic substance-induced hepatitis (Figure 2B). However, the detailed network still needs further exploration. Especially, the activation of SYK in peripheral blood mononuclear cells and inflammatory macrophages is enhanced in patients with hepatitis, suggesting the potential of non-hepatic SYK as therapeutic target (Figure 2C) [40, 41]. SYK in mononuclear cell is involved in TLR4 signaling pathway and regulates the expression and secretion of inflammatory cytokines [54]. In addition, SYK in macrophages regulates liver inflammation via crosstalking with Erk/Hif1α signaling pathway and the inhibition of SYK can remodel liver inflammatory environment and rescue liver inflammation in mouse model [16]. Recently, Carmelo Luci et al. demonstrated that an ITAM-containing adaptor, 3BP2, is also related with SYK function in steatohepatitis. SYK in myeloid cells strongly suppresses the recruitment of infiltrating neutrophils and macrophages into the liver, and either 3BP2 deficiency or SYK deletion in myeloid cells shows therapeutic effects against liver inflammation, indicating the important position of SYK-3BP2 signaling pathway in the development of liver inflammatory diseases [24].

### 3.2 SYK and liver fibrosis

Liver fibrosis is characterized by the excessive deposition of extracellular matrix (ECM) protein and the formation of liver tissue scar, which may further develop into liver cirrhosis and even hepatocellular carcinoma. Hepatic stellate cells (HSCs) are considered as the core participants in the deposition of ECM, and several studies have confirmed that the activation of HSCs can be regulated by SYK, which is increased in the development of liver fibrosis and liver cirrhosis [55, 56]. Moreover, both protein and mRNA level of SYK in HSCs and hepatocytes is positively correlated with α-SMA (the maker of liver fibrosis) in liver biopsies from patients and CCL4-induced mice liver fibrosis model. In addition, the expression of transcription factors (CBP, MPB and MYC) relevant with HSCs activation also can be up-regulated by SYK [7]. Therefore, the inhibition of SYK is essential in the treatment of liver fibrosis (Figure 2D).

Currently, some SYK inhibitors, such as GS-9973, Piceatannol and PRT062607, have been confirmed to hold important potential against liver fibrosis. For example, pharmacological inhibition of SYK using Piceatannol suppresses the activation of Caspase-1 and reduces IL-1β production in HSCs, and further releases mouse hepatic fibrosing inflammation induced by soluble egg antigen [13]. Moreover, the toxin-induced hepatic fibrosis also can be ameliorated by SYK inhibitors (Piceatannol and PRT062607), and lots of genes related with apoptosis, cell cycle and angiogenesis are affected by these small molecule inhibitors targeting SYK in HSCs [42]. Recently, researchers revealed that SYK enhances the activation of HSCs via facilitating the generation of reactive oxygen species (ROS) and autophagy in liver, and the combinational application of Silybin (ROS inhibitor) and GS-9973 (SYK inhibitor) hold promising therapeutic action against mouse liver fibrosis and liver cirrhosis [56]. However, the detailed mechanisms still need our further investigation. On the other side, non-hepatic SYK is also regarded as an important therapy target in liver fibrosis. The inhibition of SYK activity with the selective small molecule inhibitors Piceatannol and PRT062607, mitigates the acquisition of inflammatory phenotype in myeloid cells, B cells and T cells in liver. Especially, the SYK inhibition decreases the expression of TNF-α and IL-8 and suppresses mTOR signaling pathway as well as oxidative phosphorylation in myeloid cells, which protect from hepatic fibrosis effectively (Figure 2E) [42]. Collectively, these studies suggest that SYK is considered as an attractive target for hepatic fibrosis. More importantly, both hepatic SYK and non-hepatic SYK hold significant positions in the treatment of liver fibrosis.

### 3.3 SYK and hepatocellular carcinoma

The function of SYK in various cancers has been investigated intensively. However, the results are still inconsistent. For example, compared with normal tissues, the brain tumors and breast tumors hold much higher expression of SYK, while the level of SYK is lower in leukemia and lymphoma [44]. Even though lots of studies have indicated that SYK negatively regulates cell proliferation and migration in cancer cells, lots of SYK inhibitors are considered as promising anti-cancer drugs in both clinical studies and pre-clinical studies [1, 19, 57, 58]. Especially, based on the important function of SYK in hematopoietic cells, the therapeutic action of SYK inhibitors has been confirmed in multiple myeloma, such as acute myeloid leukemia and chronic lymphocytic leukemia [1, 59, 60]. In addition, the relationship between SYK and hepatocellular carcinoma has been clarified gradually in recent years. Some researchers showed that the patients with negative expression of SYK in hepatocellular carcinoma hold lower overall survival rate, compared with the patients with positive expression of SYK [58]. The full-length SYK inhibits metastasis in hepatocellular carcinoma, while the SYK-S lacking 23 amino acids in the IDB, promotes the growth and invasion of hepatocellular carcinoma. Moreover, epithelial-mesenchymal transition (EMT) also can be enhanced by SYK-S, and the expression of SYK-S is negatively correlated with overall survival rate for the patients with hepatocellular carcinoma (Figure 2F) [19, 61]. Meanwhile, scientists also noticed that the hypermethylation of CpG island in SYK promoter is related with the poor prognosis of patients. Therefore, both full-length SYK and SYK-S are regarded as diagnostic and prognostic makers in human hepatocellular carcinoma.

In addition, several groups have confirmed the therapeutic action of SYK inhibitors against hepatocellular carcinoma. For example, the inhibition of SYK induced by Piceatannol and PRT062607 also promotes the intra-tumoral expression of tumor suppressor genes (p16 and p53), but suppresses the expression of Bcl-xL and SMAD4 in hepatocarcinogenesis, showing the therapeutic action against hepatocarcinogenesis [42]. However, the detailed mechanisms are still unclear for us. Some groups investigated the upstream of SYK in hepatocellular carcinoma, and the results indicated that SYK can be regulated by checkpoint kinase 1 (CHK1) effectively, and the phosphorylation of SYK induced by CHK1 enhances the subsequent proteasomal degradation of SYK in hepatocellular carcinoma [62]. In addition, the activation of SYK also can be positively regulated by CD81, a member of the tetraspanin protein family, which promotes the development of hepatocellular carcinomas (Figure 2F). In recent years, some studies also indicated that SYK joins in the regulation of PI3K/Akt signaling pathway, which hold a key role in CX3CL1-induced platelet migration as well as cell apoptosis in human hepatocellular carcinomas [63, 64]. Totally, the accurate position of SYK in hepatocellular carcinomas is still in controversial although some SYK inhibitors have showed the therapeutic effect against human hepatocarcinogenesis. Besides, the non-hepatic SYK also hold a significant position in the treatment of liver cancer, because of the enhancement of monocyte-derived macrophages on tumor progression (Figure 2G) [65, 66, 67].

## 4 Application of small molecule SYK inhibitors in liver disease treatment

In recent years, SYK signaling pathway has been considered as a promising therapeutic target for kinds of diseases, such as inflammatory disorders, autoimmune diseases and cancers. Some SYK inhibitors (e.g. fostamatinib (R788), entospletinib (GS-9973), and cerdulatinib (PRT0620-70)) have been tested in clinical studies, even though the specificity and selectivity of different SYK inhibitors still need to be investigated and improved intensively. Meanwhile, entospletinib, one of the second generati-on SYK inhibitors, also shows promising therapeutic effect against B-cell malignancies in clinical trials, and some combination regimens based on SYK inhibitors are evaluated in several malignancies as well [9, 68, 69]. However, some side effects have been observed in the treatment as well. For example, high doses of fostamatinib causes dizziness, hypertension, neutropenia and the upregulation of serum alanine transaminase in the patients with rheumatoid arthritis, influencing the widely clinical application of such SYK inhibitor [70]. Therefore, more specific SYK inhibitors with less side effects are still of great clinical value.

Currently, some SYK inhibitors have been confirmed to hold the potential in the treatment of liver diseases (Table 1). For example, the treatment with SYK inhibitor R406 attenuates alcohol-induced liver diseases as well as non-alcoholic steatohepatitis. The phosphorylation of ERK1/2 and the activation of NFκB signaling pathway in hepatocytes are also inhibited by R406, which relieves the production of proinflammatory cytokines and mouse hepatic inflammation [40, 41, 53, 71]. Besides, another SYK inhibitor, GS-9973, also has been demonstrated to hold effective therapeutic action against mouse alcohol-induced liver injury as well as liver fibrosis by different groups [7, 16, 56, 72]. Moreover, the SYK inhibitors, Piceatannol and PRT062607, also shows the protection against toxin-induced hepatic hepatitis and fibrosis. Especially, SYK inhibition induced by Piceatannol and PRT062607 leads to the upregulation of anti-cancer genes, and also affects the expression of genes related with cell proliferation and cell apoptosis, indicating the potential of the two inhibitors in treating mouse hepatic oncogenesis [42]. However, the reports about application of SYK inhibitors against hepatocarcinogenesis are still limited, even though several SYK inhibitors have been tested in clinical trials relevant with hematological oncology. Especially, the position of SYK in hepatocellular carcinomas is still in controversial, and which SYK inhibitor holds best therapeutic action against hepatocarcinogenesis remains unclear. Thus, the best strategy to treat hepatocellular carcinomas using SYK inhibitors still needs further investigation from different groups.

**Table 1:** Application of SYK inhibitors in the treatment of liver diseases.

Inhibitor	Therapeutic action	Molecular structure	Refs.
R406	alcohol-induced liver diseases; non-alcoholic steatohepatitis		[21, 31, 33, 50]
GS-9973	alcohol-induced liver injury; liver fibrosis		[10, 12, 35, 51]
Piceatannol	toxin-induced hepatic hepatitis; liver fibrosis; hepatocellular carcinoma		[36]
PRT062607	toxin-induced hepatic hepatitis; liver fibrosis; hepatocellular carcinoma		[36]

## 5 Conclusions and perspectives

Currently, SYK is mainly considered as a promising therapeutic target for autoimmune diseases as well as hematological tumors, and lots of small molecule SYK inhibitors have been used in the treatment of various diseases in both pre-clinical studies and clinical studies. Moreover, the expression of SYK gene is also designated as a novel prognostic marker for multiple types of cancers. Some studies have demonstrated that the inhibition of SYK activity relieves the aggravation of different liver diseases (e.g. hepatitis, liver fibrosis and cirrhosis, and hepatocellular carcinomas), indicating the great therapeutic potential of SYK inhibitors in the disease treatment.

However, only four SYK inhibitors have been tested in the treatment of liver diseases, and it’s still unclear for us which inhibitor holds the best therapeutic action. Moreover, the off-target effect and related immunosuppression also limit the application of SYK inhibitors because of the increased risk of infection and side effects, and it’s also possible that the best inhibitor in various liver disease is different from each other. Therefore, it’s necessary to develop more effective and specific SYK inhibitors in the field of hepatology in the future.

Taken together, current studies highlight the significance of SYK signaling pathway in the diagnosis and treatment of liver diseases. Even though the accurate role of SYK in different liver diseases may be inconsistent, the inhibition of SYK activity induced by small molecule compounds is widely regarded as a promising therapy mode against different liver diseases. The therapeutic action of SYK inhibitors should base on the suppressing effect on both hepatic SYK and non-hepatic SYK. However, it’s still unclear for us which kind of SYK holds the major position in different liver diseases. The better understanding of SYK function will provide novel directions for the development of more reliable therapy strategies for the patients with liver diseases.

## Declarations

### Author contribution statement

All authors listed have significantly contributed to the development and the writing of this article.

### Funding statement

This study was supported by “The Young Talent Support Plan” of Xi’an Jiaotong University (For P. Liu), National Natural Science Foundation of China (31900547), Introducing overseas high-level talent intelligence projects of Xi’an City (2022JH-GCRC-0063) and Medical “Base-Clinic” Integrated Innovation Project of Xi’an Jiaotong University.

### Data availability statement

Data included in article/supp. material/referenced in article.

### Declaration of interest’s statement

The authors declare no conflict of interest.

### Additional information

No additional information is available for this paper.

## References

[1] Shao Y., Zhang S., Zhang Y., Liu Z. (2021). Recent advance of spleen tyrosine kinase in diseases and drugs. Int. Immunopharm..

[2] Mocsai A., Ruland J., Tybulewicz V.L. (2010). The SYK tyrosine kinase: a crucial player in diverse biological functions. Nat. Rev. Immunol..

[3] McAdoo S.P., Prendecki M., Tanna A., Bhatt T., Bhangal G., McDaid J. (2020). Spleen tyrosine kinase inhibition is an effective treatment for established vasculitis in a pre-clinical model. Kidney Int..

[4] Yaghini F.A., Li F., Malik K.U. (2007). Expression and mechanism of spleen tyrosine kinase activation by angiotensin II and its implication in protein synthesis in rat vascular smooth muscle cells. J. Biol. Chem..

[5] Li S., Liu R., Xue M., Qiao Y., Chen Y., Long G. (2019). Spleen tyrosine kinase-induced JNK-dependent NLRP3 activation is involved in diabetic cardiomyopathy. Int. J. Mol. Med..

[6] Gao D., Wang L., Zhang H., Yan X., Yang J., Zhou R. (2018). Spleen tyrosine kinase SYK(L) interacts with YY1 and coordinately suppresses SNAI2 transcription in lung cancer cells. FEBS J..

[7] Qu C., Zheng D.D., Li S., Liu Y.J., Lidofsky A., Holmes J.A. (2018). Tyrosine kinase SYK is a potential therapeutic target for liver fibrosis. Hepatology.

[8] Pamuk O.N., Tsokos G.C. (2010). Spleen tyrosine kinase inhibition in the treatment of autoimmune, allergic and autoinflammatory diseases. Arthritis Res. Ther..

[9] Liu D., Mamorska-Dyga A. (2017). Syk inhibitors in clinical development for hematological malignancies. J. Hematol. Oncol..

[10] Suljagic M., Longo P.G., Bennardo S., Perlas E., Leone G., Laurenti L. (2010). The Syk inhibitor fostamatinib disodium (R788) inhibits tumor growth in the Emu- TCL1 transgenic mouse model of CLL by blocking antigen-dependent B-cell receptor signaling. Blood.

[11] Currie K.S., Kropf J.E., Lee T., Blomgren P., Xu J., Zhao Z. (2014). Discovery of GS-9973, a selective and orally efficacious inhibitor of spleen tyrosine kinase. J. Med. Chem..

[12] Coffey G., Betz A., DeGuzman F., Pak Y., Inagaki M., Baker D.C. (2014). The novel kinase inhibitor PRT062070 (Cerdulatinib) demonstrates efficacy in models of autoimmunity and B-cell cancer. J. Pharmacol. Exp. Therapeut..

[13] Lu Y.Q., Zhong S., Meng N., Fan Y.P., Tang W.X. (2017). NLRP3 inflammasome activation results in liver inflammation and fibrosis in mice infected with Schistosoma japonicum in a Syk-dependent manner. Sci. Rep..

[14] van Bree S.H., Gomez-Pinilla P.J., van de Bovenkamp F.S., Di Giovangiulio M., Farro G., Nemethova A. (2013). Inhibition of spleen tyrosine kinase as treatment of postoperative ileus. Gut.

[15] Hu Q., Liu M., You Y., Zhou G., Chen Y., Yuan H. (2021). Dual inhibition of reactive oxygen species and spleen tyrosine kinase as a therapeutic strategy in liver fibrosis. Free Radic. Biol. Med..

[16] Chen X., Wang Z., Han S., Wang Z., Zhang Y., Li X. (2021). Targeting SYK of monocyte-derived macrophages regulates liver fibrosis via crosstalking with Erk/Hif1alpha and remodeling liver inflammatory environment. Cell Death Dis..

[17] Westbroek M.L., Geahlen R.L. (2017). Modulation of BCR signaling by the induced dimerization of receptor-associated SYK. Antibodies (Basel).

[18] Latour S., Zhang J., Siraganian R.P., Veillette A. (1998). A unique insert in the linker domain of Syk is necessary for its function in immunoreceptor signalling. EMBO J..

[19] Hong J., Yuan Y., Wang J., Liao Y., Zou R., Zhu C. (2014). Expression of variant isoforms of the tyrosine kinase SYK determines the prognosis of hepatocellular carcinoma. Cancer Res..

[20] Wang L., Duke L., Zhang P.S., Arlinghaus R.B., Symmans W.F., Sahin A. (2003). Alternative splicing disrupts a nuclear localization signal in spleen tyrosine kinase that is required for invasion suppression in breast cancer. Cancer Res..

[21] Zhang Y., Oh H., Burton R.A., Burgner J.W., Geahlen R.L., Post C.B. (2008). Tyr130 phosphorylation triggers Syk release from antigen receptor by long-distance conformational uncoupling. Proc. Natl. Acad. Sci. U. S. A.

[22] Wang L., Aschenbrenner D., Zeng Z., Cao X., Mayr D., Mehta M. (2021). Gain-of-function variants in SYK cause immune dysregulation and systemic inflammation in humans and mice. Nat. Genet..

[23] Nabhan M., Legrand F.X., Le-Minh V., Robin B., Bechara R., Huang N. (2020). The FcgammaRIIa-Syk Axis controls human dendritic cell activation and T cell response induced by Infliximab aggregates. J. Immunol..

[24] Luci C., Vieira E., Bourinet M., Rousseau D., Bonnafous S., Patouraux S. (2022). SYK-3BP2 pathway activity in parenchymal and myeloid cells is a key pathogenic factor in metabolic steatohepatitis. Cell Mol. Gastroenterol. Hepatol..

[25] Eipel C., Abshagen K., Vollmar B. (2010). Regulation of hepatic blood flow: the hepatic arterial buffer response revisited. World J. Gastroenterol..

[26] Tanabe K., Taura K., Koyama Y., Yamamoto G., Nishio T., Okuda Y. (2015). Migration of splenic lymphocytes promotes liver fibrosis through modification of T helper cytokine balance in mice. J. Gastroenterol..

[27] Li L., Duan M., Chen W., Jiang A., Li X., Yang J. (2017). The spleen in liver cirrhosis: revisiting an old enemy with novel targets. J. Transl. Med..

[28] Swarup G., Dasgupta J.D., Garbers D.L. (1983). Tyrosine protein kinase activity of rat spleen and other tissues. J. Biol. Chem..

[29] Yue F., Cheng Y., Breschi A., Vierstra J., Wu W., Ryba T. (2014). A comparative encyclopedia of DNA elements in the mouse genome. Nature.

[30] Tarantino G., Citro V., Balsano C. (2021). Liver-spleen axis in nonalcoholic fatty liver disease. Expet Rev. Gastroenterol. Hepatol..

[31] Barrea L., Di Somma C., Muscogiuri G., Tarantino G., Tenore G.C., Orio F. (2018). Nutrition, inflammation and liver-spleen axis. Crit. Rev. Food Sci. Nutr..

[32] Xie X.L., Liu X., Ou J. (2019). Is synchronous hepatectomy and splenectomy superior to hepatectomy alone for selected patients with hepatocellular carcinoma and clinically significant portal hypertension?. J. Surg. Oncol..

[33] Shi R., Zhang Y.M., Zhu Z.J., Deng Y.L., Pan C., Zheng H. (2014). Synchronous splenectomy and hepatectomy in patients with hepatocellular carcinoma, hypersplenism and liver cirrhosis. Hepato-Gastroenterology.

[34] Zheng Z., Wang H., Li L., Zhang S., Zhang C., Zhang H. (2020). Splenectomy enhances the Ly6C(low) phenotype in hepatic macrophages by activating the ERK1/2 pathway during liver fibrosis. Int. Immunopharm..

[35] Jiang W., Li Y., Wei W., Li J.W., Li L., Zhang C. (2020). Spleen contributes to restraint stress induced hepatocellular carcinoma progression. Int. Immunopharm..

[36] Liang Q.S., Xie J.G., Yu C., Feng Z., Ma J., Zhang Y. (2021). Splenectomy improves liver fibrosis via tumor necrosis factor superfamily 14 (LIGHT) through the JNK/TGF-beta1 signaling pathway. Exp. Mol. Med..

[37] Shand F.H., Ueha S., Otsuji M., Koid S.S., Shichino S., Tsukui T. (2014). Tracking of intertissue migration reveals the origins of tumor-infiltrating monocytes. Proc. Natl. Acad. Sci. U. S. A.

[38] Petty A.J., Owen D.H., Yang Y., Huang X. (2021). Targeting tumor-associated macrophages in cancer immunotherapy. Cancers.

[39] Duan Z., Luo Y. (2021). Targeting macrophages in cancer immunotherapy. Signal Transduct. Targeted Ther..

[40] Kurniawan D.W., Jajoriya A.K., Dhawan G., Mishra D., Argemi J., Bataller R. (2018). Therapeutic inhibition of spleen tyrosine kinase in inflammatory macrophages using PLGA nanoparticles for the treatment of non-alcoholic steatohepatitis. J. Contr. Release.

[41] Bukong T.N., Iracheta-Vellve A., Saha B., Ambade A., Satishchandran A., Gyongyosi B. (2016). Inhibition of spleen tyrosine kinase activation ameliorates inflammation, cell death, and steatosis in alcoholic liver disease. Hepatology.

[42] Torres-Hernandez A., Wang W., Nikiforov Y., Tejada K., Torres L., Kalabin A. (2019). Targeting SYK signaling in myeloid cells protects against liver fibrosis and hepatocarcinogenesis. Oncogene.

[43] Sun Y., Yu H.T., Li F.F., Lan L.Q., He D.X., Zhao H.J. (2020). Identification of hub genes and potential molecular mechanisms in patients with HBV-associated acute liver failure. Evol. Bioinf. Online.

[44] Alwithenani A.I., Althubiti M.A. (2020). Systematic analysis of spleen tyrosine kinase expression and its clinical outcomes in various cancers. Saudi J. Med. Med. Sci..

[45] Kurniawan D.W., Storm G., Prakash J., Bansal R. (2020). Role of spleen tyrosine kinase in liver diseases. World J. Gastroenterol..

[46] Bukong T.N., Kodys K., Szabo G. (2014). A novel human Radixin peptide inhibits hepatitis C virus infection at the level of cell entry. Int. J. Pept. Res. Therapeut..

[47] Xue Y.H., Bhushan B., Mars W.M., Bowen W., Tao J.Y., Orr A. (2020). Phosphorylated Ezrin (Thr567) regulates Hippo pathway and yes-associated protein (Yap) in liver. Am. J. Pathol..

[48] Bukong T.N., Kodys K., Szabo G. (2013). Human ezrin-Moesin-Radixin proteins modulate hepatitis C virus infection. Hepatology.

[49] Xue Y.H., Mars W.M., Bowen W., Singhi A.D., Stoops J., Michalopoulos G.K. (2018). Hepatitis C virus Mimics effects of glypican-3 on CD81 and promotes development of hepatocellular carcinomas via activation of Hippo pathway in hepatocytes. Am. J. Pathol..

[50] Inubushi S., Nagano-Fujii M., Kitayama K., Tanaka M., An C., Yokozaki H. (2008). Hepatitis C virus NS5A protein interacts with and negatively regulates the non-receptor protein tyrosine kinase Syk. J. Gen. Virol..

[51] Li M., Wu C., Guo H., Chu C., Hu M., Zhou C. (2019). Mangiferin improves hepatic damage-associated molecular patterns, lipid metabolic disorder and mitochondrial dysfunction in alcohol hepatitis rats. Food Funct..

[52] Tornai D., Furi I., Shen Z.T., Sigalov A.B., Coban S., Szabo G. (2019). Inhibition of triggering receptor expressed on myeloid cells 1 ameliorates inflammation and macrophage and neutrophil activation in alcoholic liver disease in mice. Hepatol. Commun..

[53] Bukong T.N., Iracheta-Vellve A., Gyongyosi B., Ambade A., Catalano D., Kodys K. (2016). Therapeutic benefits of spleen tyrosine kinase inhibitor administration on binge drinking-induced alcoholic liver injury, steatosis, and inflammation in mice. Alcohol Clin. Exp. Res..

[54] Yi Y.S., Son Y.J., Ryou C., Sung G.H., Kim J.H., Cho J.Y. (2014). Functional roles of Syk in macrophage-mediated inflammatory responses. Mediat. Inflamm..

[55] Modol T., Natal C., Perez de Obanos M.P., Domingo de Miguel E., Iraburu M.J., Lopez-Zabalza M.J. (2011). Apoptosis of hepatic stellate cells mediated by specific protein nitration. Biochem. Pharmacol..

[56] Hu Q.T., Liu M.Y., You Y.D., Zhou G., Chen Y., Yuan H. (2021). Dual inhibition of reactive oxygen species and spleen tyrosine kinase as a therapeutic strategy in liver fibrosis. Free Radic. Biol. Med..

[57] Lamb D.J., Rust A., Rudisch A., Gluxam T., Harrer N., Machat H. (2020). Inhibition of SYK kinase does not confer a pro-proliferative or pro-invasive phenotype in breast epithelium or breast cancer cells. Oncotarget.

[58] Yuan Y., Wang J., Li J., Wang L., Li M., Yang Z. (2006). Frequent epigenetic inactivation of spleen tyrosine kinase gene in human hepatocellular carcinoma. Clin. Cancer Res..

[59] Wang S., Ma Y., Wang X., Jiang J., Zhang C., Wang X. (2019). IL-17A increases multiple myeloma cell viability by positively regulating Syk expression. Transl. Oncol..

[60] Weisberg E., Meng C., Case A.E., Tiv H.L., Gokhale P.C., Buhrlage S.J. (2020). Effects of the multi-kinase inhibitor midostaurin in combination with chemotherapy in models of acute myeloid leukaemia. J. Cell Mol. Med..

[61] Shin S.H., Lee K.H., Kim B.H., Lee S., Lee H.S., Jang J.J. (2014). Downregulation of spleen tyrosine kinase in hepatocellular carcinoma by promoter CpG island hypermethylation and its potential role in carcinogenesis. Lab. Invest..

[62] Hong J., Hu K., Yuan Y., Sang Y., Bu Q., Chen G. (2012). CHK1 targets spleen tyrosine kinase (L) for proteolysis in hepatocellular carcinoma. J. Clin. Invest..

[63] Miao S., Lu M., Liu Y., Shu D., Zhu Y., Song W. (2020). Platelets are recruited to hepatocellular carcinoma tissues in a CX3CL1-CX3CR1 dependent manner and induce tumour cell apoptosis. Mol. Oncol..

[64] Jia X., Mo Z., Zhao Q., Bao T., Xu W., Gao Z. (2020). Transcriptome alterations in HepG2 cells induced by shRNA knockdown and overexpression of TMEM2 gene. Biosci. Biotechnol. Biochem..

[65] Lee H.W., Choi H.J., Ha S.J., Lee K.T., Kwon Y.G. (2013). Recruitment of monocytes/macrophages in different tumor microenvironments. Biochim. Biophys. Acta.

[66] Chen Y., Song Y., Du W., Gong L., Chang H., Zou Z. (2019). Tumor-associated macrophages: an accomplice in solid tumor progression. J. Biomed. Sci..

[67] Qian B.Z., Pollard J.W. (2010). Macrophage diversity enhances tumor progression and metastasis. Cell.

[68] Singh R., Masuda E.S., Payan D.G. (2012). Discovery and development of spleen tyrosine kinase (SYK) inhibitors. J. Med. Chem..

[69] Thoma G., Veenstra S., Strang R., Blanz J., Vangrevelinghe E., Berghausen J. (2015). Orally bioavailable Syk inhibitors with activity in a rat PK/PD model. Bioorg. Med. Chem. Lett..

[70] Deng G.M., Kyttaris V.C., Tsokos G.C. (2016). Targeting Syk in autoimmune rheumatic diseases. Front. Immunol..

[71] Fang X., Zaman M.H., Guo X., Ding H., Xie C., Zhang X. (2018). Role of hepatic deposited immunoglobulin G in the pathogenesis of liver damage in systemic lupus erythematosus. Front. Immunol..

[72] Kim J.W., Roh Y.S., Jeong H., Yi H.K., Lee M.H., Lim C.W. (2018). Spliceosome-associated protein 130 exacerbates alcohol-induced liver injury by inducing NLRP3 inflammasome-mediated IL-1beta in mice. Am. J. Pathol..

